# Achieving sustainable agricultural production under farmer conditions in maize-gliricidia intercropping in Salima District, central Malawi

**DOI:** 10.1016/j.heliyon.2019.e02632

**Published:** 2019-10-18

**Authors:** Harrington Nyirenda

**Affiliations:** Salima Agricultural Development Division, Land Resource Conservation Department, Private Bag 1, Salima, Malawi

**Keywords:** Agricultural science, Environmental science, Biological sciences, Earth sciences, Social sciences, Organic matter, Gestation period, Yield trend, Malawi

## Abstract

Smallholder farmers in Malawi are faced with limited options for climate smart agriculture that would restore soil fertility and increase maize yield. Ten plots of maize intercropped with *Gliricidia sepium* (MIG) and 10 traditional sole-maize (TSM) plots (0.2 ha each) were studied under farmer conditions from 2013/14 to 2017/18 in Salima District, central Malawi. The aim was to assess performance of MIG on soil fertility restoration and maize yield in degraded agricultural land. *G. sepium* trimmings were incorporated in MIG in October, January and September of every season. A total of 92 kg N ha-1 was applied in both treatments. Soil and maize yield measurements were done from 10 m x 10 ridges centre of each plot and a paired t-test in R Statistical Software was used for data analysis. Organic matter (p < 0.001) and nitrogen (p < 0.011) were significantly higher in MIG than in TSM while bulk density was significantly lower (p < 0.006) in MIG than in TSM. Higher maize yield was achieved in MIG (5.52 t/ha) than in TSM (1.48 t ha-1) (p < 0.001). Nonsignificant differences between MIG and TSM fields for potassium (p > 0.678) and phosphorus (p > 0.149) suggests that the nutrients were not affected by presence or absence of *G. sepium* and may not have contributed to differences in yields. Effective gestation period for maize-gliricidia intercropping was at least two years where significant maize yields were first achieved. The findings in MIG provide farmers with sustainable agricultural option for soil health renewal and maize yield increase in central Malawi.

## 1 Introduction

Developing countries including those in Africa will be the most affected by the impacts of climate change, increase in population, reduced land cover and the associated unsustainable land use (Kertesz, 2009). In Malawi, the departments of Land Resources Conservation, Forestry and Non-governmental Organisations have been implementing practices aimed at restoring degraded land through managing different agricultural ecosystems, improving biodiversity and natural forest ecosystems. The most common practices are those of Climate Smart Agriculture (CSA) (Republic of Malawi, 2017). CSA practices are premised on three major principles; improved and increased agriculture outputs, improved environment and community resilience, and thirdly enhancing carbon sequestration (Food and Agriculture Organisation of the Unites Nations, 2013; Nyirenda et al., 2019). In Malawi, the main CSA practises are agroforestry, conservation agriculture (CA), afforestation, System of Rice Intensification (SRI) and manure utilisation (Kaczan et al., 2013). These practices improve ecological systems, increase agricultural yields, reduce soil erosion and loss of nutrients. They improve agricultural sustainability by restoring degraded soils (Powlson et al., 2016). The consistent practice of CSA measures contributes to general soil health with pronounced effect on increase on soil organic carbon (Food and Agriculture Organisation of the United Nations, 2013; Powlson et al., 2016; Khatri-Chhetri et al., 2017).

Agriculture productivity in Malawi is highly dependent on climate, as it is rainfed (Sanchez, 2002; Government of Malawi, 2012). In a World Bank analysis cited by Kaczan et al. (2013), it was reported that between 1960 and 2006, the mean annual temperature had increased by over 0.9 °C especially in summer periods. In addition, the rainfall would continue to be unpredictable and inconsistent.

The increasing population in Malawi is posing a great demand on natural resources that has resulted in land degradation (Government of Malawi, 2012). Intensive use of land has led to degradation and reduced productivity (Denning et al., 2009) that resulted in soil degradation which was equivalent to agricultural production loss of MK7.5 billion ($54 million), representing a 1.6% loss from the Gross Domestic Product (Yaron et al., 2011). Another study by Chigowo (2007), estimated soil nutrient loss at an equivalent of $2 million per annum. Walker and Desanker (2004) previously proposed a timely reaction for interventions to control the loss of soil nutrients with emphasis on general soil improvement practices. While there has been re-emphasis to promote Climate Smart Agriculture (CSA) approaches like agroforestry to offset deforestation, there is also need to understand and tackle social aspects to better address environmental and land restoration programmes. In their review of agroforestry in Malawi and Conservation Agriculture in Zambia, Kaczan et al. (2013) proposed further research studies on the impact of agroforestry on the physical and chemical soil status of landscapes. It is on this basis that this study was conducted to assess the impact of selected agroforestry technologies on soil condition and maize yield in Salima District, central Malawi.

Maize production is common among smallholder farmers in Malawi (Ministry of Agriculture, Irrigation and Water Development, 2017). Maize is a staple food for about 75% of the country's population. An average of 1.7 million ha of the country's 10 million ha are used for maize production, From 2006 to 2018, the yield ranged from 2.6 to 4 million tons. Smallholder farmers produce about 3.2 t ha^−1^. Most farmers grow maize as a sole crop in the field. Maize production is traditionally associated with use of inorganic fertilizer of NPK and UREA for maize yield improvement. One bag (50 kg) of each fertilizer type is applied on 0.4 ha. Some farmers apply organic manure for soil fertility improvement while others manage naturally growing soil fertility improvement tree species like *Philenoptera violacea* and *Faidherbia albida* where maize is grown (Beedy et al., 2015). Other farmers grow maize under conservation agriculture, a system that focusses on minimum soil disturbance, maximum soil cover and crop rotations/associations (FAO, 2013; Powlson et al., 2016).

This research intended to evaluate the impact of agroforestry interventions on soil nutrients and crop yield. Agroforestry was chosen because it has been practiced for a long time in the study area (Ngwira et al., 2012) and therefore, provides good base for assessment. Specifically, one of the multi-purpose tree species in agroforestry practices is *Gliricidia sepium*. There have not been specific studies for assessing maize-gliricidia intercropping under farmer conditions in many parts of Malawi. Most information is based on such studies under research station conditions like those of Chirwa et al. (2003), Akinnifesi et al. (2006) and Makumba et al. (2006). The on-farm trials were limited to few selected farmers located around the Research station in the Shire Highlands region of Malawi. This present study was conducted as part of the on-farm scaling up of agroforestry technologies among the rural farmers, while also promoting the technology as part of Climate Smart Agriculture. The aim of the study was to assess the impact of *Gliricidia sepium* on soil after a five-season cropping cycle in Salima District, central Malawi. The specific objectives were the following: 1) to compare soil nutrient levels in maize (*Zea mays*) intercropped with *Gliricidia sepium* (MIG) and traditional sole-maize (TSM) fields; and 2) to quantify maize yield and trend in maize intercropped with *G. sepium* and traditional sole-maize fields. The study posed the following hypotheses:

H0: There is no difference in soil nutrient levels in maize intercropped with *Gliricida sepium* and traditional sole-maze fields.

H1: There is a difference in soil nutrient levels in maize intercropped with *Gliricida sepium* and traditional sole-maize fields.

H0: There is no difference in maize yield in maize intercropped with *Gliricida sepium* and traditional sole-maize fields.

H1: There is a difference in maize yield in maize intercropped with *Gliricida sepium* and traditional sole-maize fields.

## 2 Materials and methods

### 2.1 Study area

This research project was conducted in Salima District, central Malawi. Salima District is among the top six districts in the country that are very vulnerable to climate change impacts (Government of Malawi, 2006). It is also prone to floods and droughts. The CSA interventions implemented are expected to reduce the impacts of climate change. According to Malawi Meteorological Office (unpublished data), three major seasons characterise the study district: cool dry season (May to August), warm wet season (November to April) and hot dry season (September to October), annual rainfall averaging 860 mm–1400 mm. Rainfall for the period under study is shown in Table 1. The rainfall is unimodal characterised with unpredictable variability falling from November to April. The average maximum monthly temperature is 28.7 °C and minimum temperature of 20 °C. October is the warmest while July is the coolest month. The dominant vegetation is Miombo woodland. The forest woodlands in the area are dominated by tree species like *Pterocarpus rotundifolius, Combretum sp*, *Bauhinia petersiana, Lannea discolor* while the agricultural land is dominated by *Faidherbia albida*, *Philenoptera violacea Stereospermum kunthianum* (Nyirenda et al., 2019). Major agricultural crops include maize (*Zea mays)*, cassava (*Manihot esculenta*) as food crops; and tobacco, rice, ground nut (*Arachis hypoaea*), cotton (*Gossypium hirsutum*) as cash crops. Minor production of chillies, soya (*Glycine max*), sorghum (*Sorghum bicolor*), cowpeas (*Vigna unguiculata*), and sweet potato (*Ipomoea batatas*) is also practised.

**Table 1:** Rainfall amounts during the season under study in Salima District, central Malawi.

Season	2013/14	2014/15	2015/6	2016/17	2017/18
Rainfall (mm)	1116.2	918.6	995.4	1114.3	1025.4

Source: Malawi Meteorological Office, 2018 (unpublished data).

### 2.2 Study design and data collection

The study had two treatments as follows:

i. Traditional sole maize crop production (TSM) as a control-where no agroforestry is practiced
ii. Maize intercropped with *Gliricida sepium* (MIG) as an agroforestry practice

Ten plots of TSM and MIG each were set as field laboratories in 2012 under a government-funded project on sustainable agriculture. Each TSM plot has its adjacent MIG plot. The plots are 0.2 ha each. Ridges were made on same place in the MIG but shifted in the TSM. In the MIG, one seedling of *G. sepium* was planted per station at 1.8 m × 90 cm (jump one fallow); giving 6,173 tree seedlings ha^−1^. The *G. sepium* was first planted in 2012/13 season to make sure that it was used in 2013/14 season while the maize was not planted in both treatments in 2012/13. In October of every season, the *G. sepium* plants were trimmed at 30 cm height and all materials buried under ridge not more than 15 cm deep (Akinnifesi et al., 2006). This incorporation coincided with start of rains. A second pruning of *G. sepium* was done in January in which trimmings were not buried to avoid root damage for maize and *G. sepium*. For second trimming, the material/biomass were placed along the ridge. The observed height for *G. sepium* before the first trimming in first season (2013/14) was about 180 cm. In the subsequent seasons, the observed average height before January and October trimming was 45 cm and 121 cm respectively. The amount of trimmed and incorporated *G. sepium* materials was not strictly measured in this study because the focus was to observe changes in soil nutrients and maize yield rather than the productivity of *G. sepium* itself. The other reason was that the emphasis was on ensuring that the treatments were implemented under farmers’ convenient condition where strict biomass measurements as a practice is rare. However, this was cautiously noted that it may have resulted in over or under application of these trimmings and have a bearing on crop yield. Maize variety DK 8053 was planted about 14 days after the October incorporation. Traditionally, farmers applied 92 kg Nitrogen (N) ha^−1^ of inorganic fertilizer nitrogen each in both treatments. So far, the practice has been done for five seasons and this assessment covers this duration. Appropriate weeding schedule was followed. Only the central part of the fields of 10m by 10 ridges of maize was harvested for assessment on yield measurements, soil nutrients and bulk density. The maize grain yield was taken at 13% of moisture content (Akinnifesi et al., 2006).

Ten soil samples were collected from each treatment. Four points were identified in each plot where the harvesting for yield measurement was done and soil was collected and mixed to come up with a composite sample of about 1 kg that was taken for analysis for soil nutrient status. Samples for bulk density were collected separately using cores (Brady and Weil, 1996). The soil nutrients under focus included: nitrogen (N), organic matter (OM), soil organic carbon (SOC), considered most limiting in Malawian soil (Njoloma et al., 2016), phosphorus (P) and potassium (K). The samples were collected at a depth of 0–15 cm, considered as fertile part (Brady and Weil, 1996) using a 4.5 cm diameter soil auger. The samples were put in plastic packets and sealed during delivery to the laboratory. The data for yield was taken from the annual records available by treatment host farmers.

### 2.3 Soil and data analysis

Soil samples were analysed at Chitedze Agricultural Research Station. Soil texture was analysed using hydrometer method (United States Department of Agriculture, 1954; Gee and Bauder, 1979) while bulk density by procedures provided by Brady and Weil (1999). The soil pH was measured using pH meter (Kalra, 1995). Total soil carbon content was determined by following Walkley-Black procedures (Nelson and Sommers, 1982; Schumacher, 2002). Total nitrogen content was analysed by Kjeldal method (Bremner and Keeney, 1966) while phosphorus by Bray and Kurtz No. 1 extractant method (Bray and Kurtz, 1945), and potassium by flame photometer reading. Organic matter was analysed using dichromate oxidation method (Kalra, 1995).

Soil parameters and yield data were analysed using R Statistical Software (R Development Core Team, 2017). Data were tested for homoscedasticity and normality using Shapiro-Wilk normality test and Levene test respectively (Assede et al., 2015) to ensure the data met statistical conditions. The tested parameters met either normality, homoscedasticity or both, therefore, no data transformation was done. A paired t-test was computed to compare significant differences between soil nutrient levels and maize yields in the two treatments. The significant level was taken at p < 0.05.

## 3 Results

### 3.1 Soil nutrient and bulk density dynamics

**Fig. 1:**
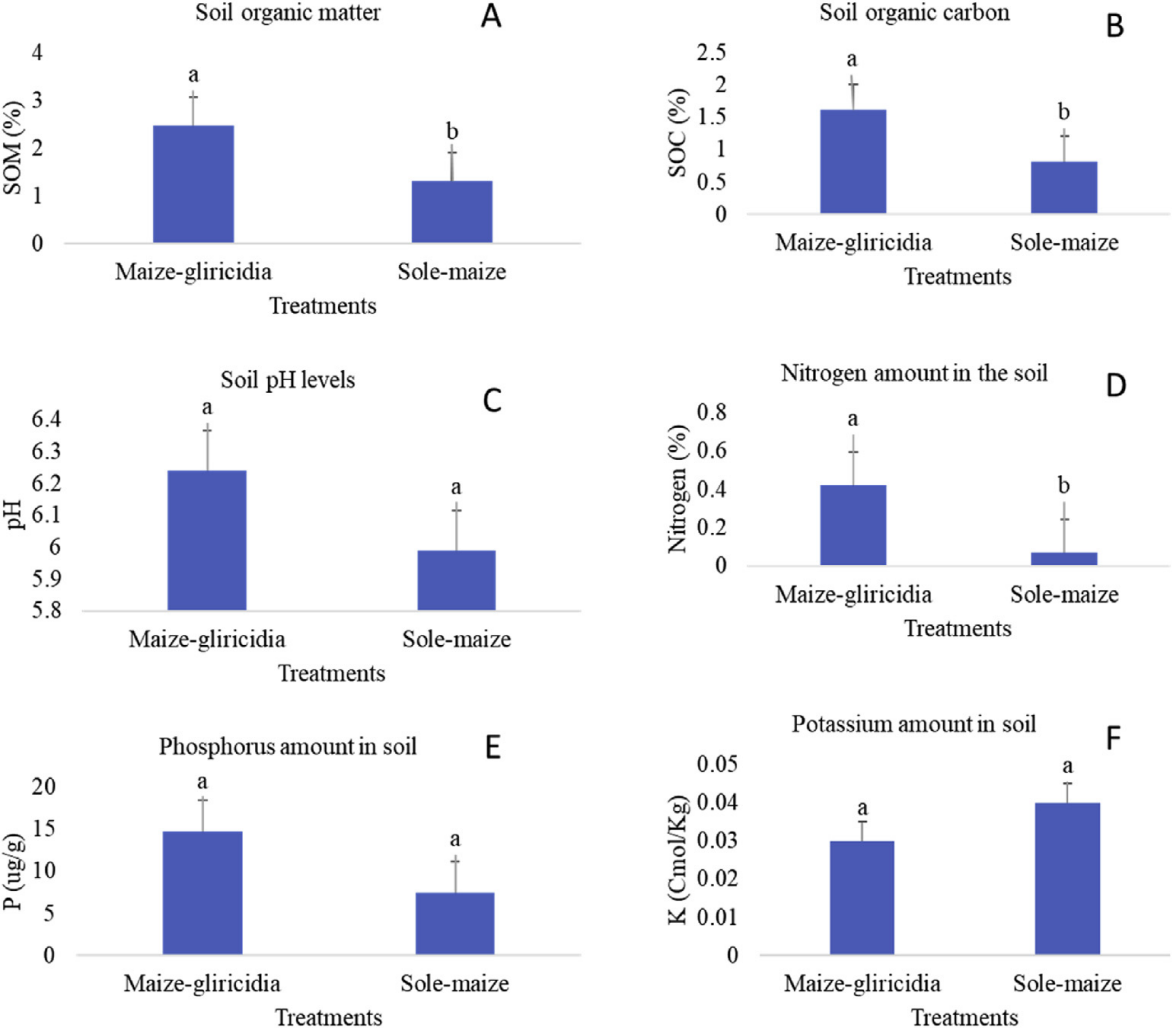
Soil nutrient comparison in sole-maize field and in maize-gliricidia intercropping in Salima District, central Malawi (A = % Soil organic matter, B = % Soil organic carbon, C = Soil pH, D = % Nitrogen, E = Phosphorus, F = Potassium).

**Fig. 2:**
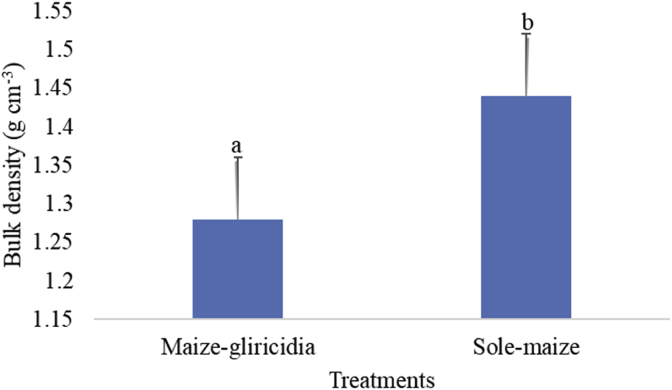
Soil bulk density comparison in sole-maize field and in maize-gliricidia intercropping field in Salima District, central Malawi.

In this study where it was hypothesised that there is no difference in soil nutrient levels in maize intercropped with *Gliricida sepium* and traditional sole-maize fields; and that there is no difference in maize yield in maize intercropped with *Gliricida sepium* and traditional sole-maize fields. It was observed that the practice of maize-gliricidia intercropping in Salima, central Malawi improves soil fertility especially for the most limiting nutrients of soil organic carbon, nitrogen, and organic matter. The intercropping of maize with *G. sepium* for five seasons has provided indications that improved soil fertility would be achieved (Fig. 1). The MIG had significantly (p < 0.001) higher OM than the TSM field. There was also significantly (p < 0.001) increased N in the MIG compared to the TSM. There was no significant difference between MIG and TSM fields for soil pH (p > 0.356), potassium (p > 0.678) and phosphorus (p > 0.149). The MIG plots had significantly (p < 0.006) lower bulk density than in the TSM field with actual values being 1.28 g cm^−3^ and 1.44 g cm^−3^, respectively (Fig. 2).

### 3.2 Yield amount and trend

**Fig. 3:**
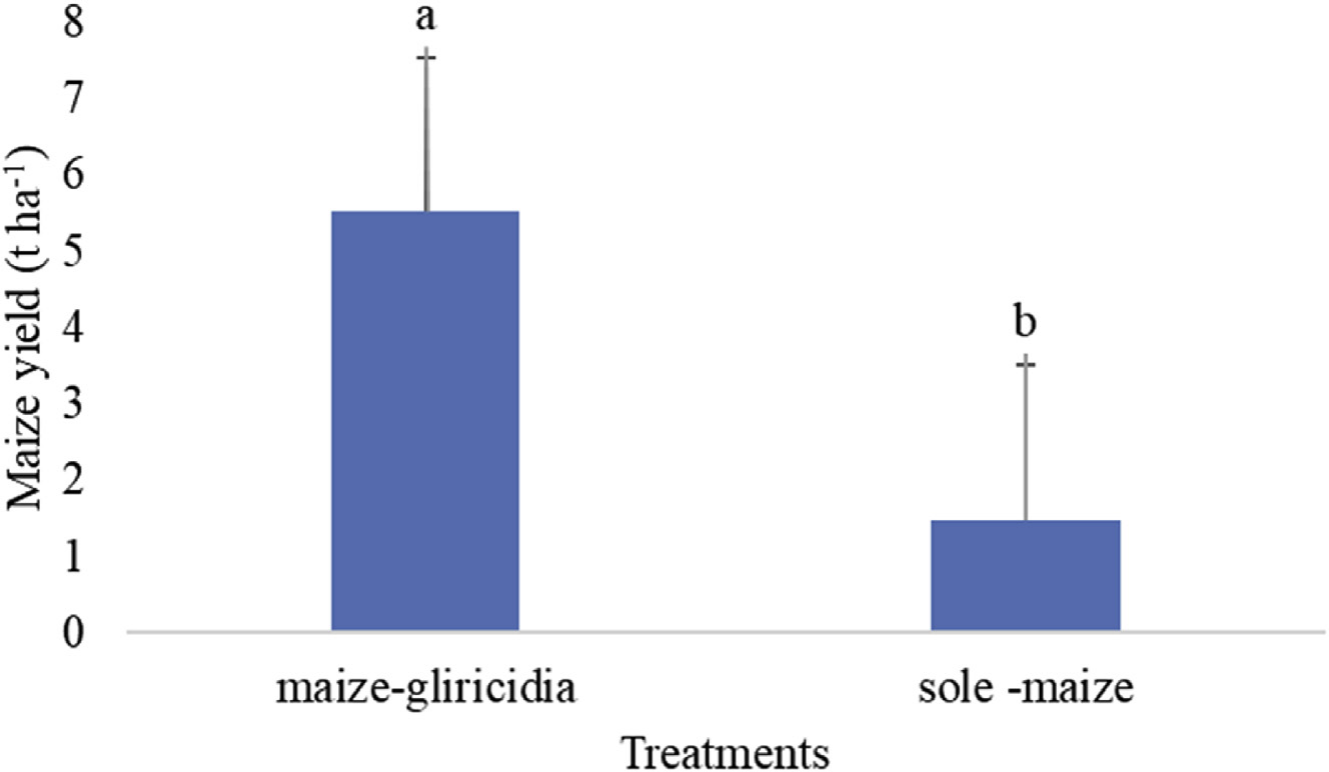
Maize yield comparison in sole-maize field and maize-gliricidia intercropping field in Salima District, central Malawi.

**Fig. 4:**
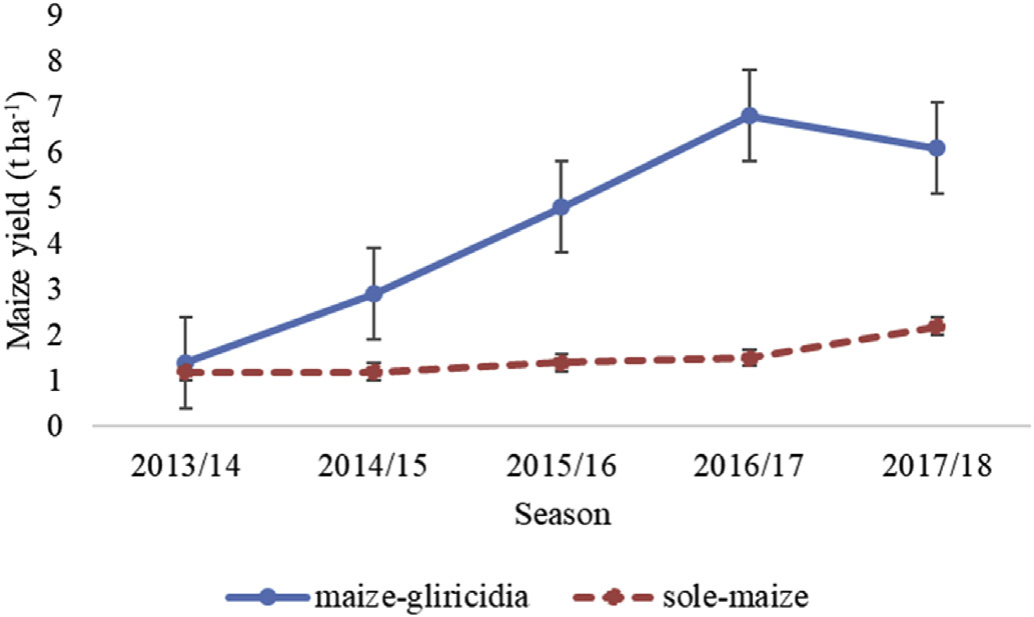
Maize yield trend in sole-maize field and maize-gliricidia-intercropping field in five-season period in Salima District, central Malawi.

Maize yield improved more in MIG than TSM. MIG is therefore**,** a sustainable way to maize production in central Malawi. In the fifth season, the maize yield (Fig. 3) was significantly (p < 0.001) higher (5.52 t ha^−1^) in MIG than in the TSM (1.48 t ha^−1^) field. Over the five seasons, the annual trend (Fig. 4) for yield showed no statistical difference in the first season (p > 0.326). However, the significant difference was noticed from second season (p < 0.001) to the fifth season (Fig. 4). The trend shows that major differences in yield peaked from third year. The highest yield was recorded in 2016/17 season (6.8 t ha^−1^).

## 4 Discussion

### 4.1 Soil nutrient and bulk density changes

Returning plant residues to the soil increases amount of nutrients available. Crops return 25% nitrogen and phosphorus, 50% sulphur, 75% potassium out of the nutrients it takes hence retuning them is essential (Sarkar, 2015). Different agroforestry species affect the nutrient cycle differently. For example, shade species affect nutrient cycle differently because of variations in biomass of aboveground, decomposition rates and root biomass (Nair and Latt, 1997). These findings support the fact that nitrogen fixed by shady trees could be transferred to below ground and accessed by non-nitrogen fixing tree or crops like maize. The increased organic matter, organic carbon and nitrogen in the MIG indicates that *G. sepium* facilitated nutrient build up. The nutrient improvement status meant general soil health enhancement thereby enabling yield response for maize. Akinnifesi et al. (2006), recorded improved soil fertility in maize-gliricidia field compared to sole-maize in a 10-year period in southern Malawi. The pruned materials provided nutrients content of about 12 Mg K g^−1^, 4 Mg P g^−1^ and 29 Mg N g^−1^ from 1993 to 1998. They reported higher SOC (3 g kg^−1^) in maize-*G. sepium* in the 0–20 cm depth than in sole-maize but this reduced with increasing depth. *G. sepium* is beneficial in soil profile nutritional cycling due to its deep root system than seasonal trees (Nair et al., 1995).

The soils in both treatments had low bulk density (<1.6 g cm^−3^) implying that there was no compaction to hinder root development (Mckenzie et al., 2004). However, high levels of OC and OM in MIG might have been responsible for its relatively lower bulk density compared to that of TSM. Lower OC and OM is associated with higher bulk density (Arnalds et al., 2013; Chaudhari et al., 2013; Arnalds, 2015). In this study, it can be hypothesized that the annual decomposing Gliricidia trimmings might have increased OM as observed by Zheng et al. (2017) thereby influencing bulk density.

The not significant difference in both treatments for phosphorus and potassium may mean that the elements were maintained by the *G. sepium* and inorganic fertilizer application, that is, the amounts taken by the crops were like those supplied by *G. sepium* (Makumba et al., 2006). This may also suggest that the elements may not have contributed much to differences in yields in the two treatments. The burning, as part of land preparation in TSM might have accumulated potash (Pachon et al., 2013). General management and the use of *G. sepium* may have increased phosphorus amounts (Ngwira et al., 2012) as some leguminous crops increase phosphorus availability in soil (Li et al., 2007). Furthermore, phosphorus is rarely mobile and only 10–30% is used in the season of application and the rest is used in later seasons (Li et al., 2007), making it almost equally available in both treatments.

### 4.2 Yield status

The lack of significant difference in maize yield in both treatments in first year could be due to low levels of OM, OC and N situation in MIG treatment. The higher maize yield from second season in MIG to fifth season compared to TSM could be because of nutrient build-up from *Gliricidia sepium* trimmings. Chirwa et al. (2006) reported that sole-maize field without application of inorganic fertilizer registered high yield in first year and reduced in subsequent years. This means that inorganic fertilizer in TSM just helped maintain maize yield production but did not improve soil productivity. The addition of trimmings sustained increased maize yield in MIG where continuous maize growing in TSM without organic additions might have led to nutrient depletion in the soil resulting in low maize yields. Akinnifesi et al. (2006) reported significantly higher maize yield in maize-gliricidia than in sole-maize fields from second year of the experiment. They recorded an average yield of 3.8 t/ha in maize-gliricidia field and 1.2 t/ha in sole-maize field for NSCM 41 maize variety after 11 years. However, on yearly basis, the yield could reach 7.1 t/ha. The present study recorded an average yield of 5.52 t ha^−1^ in MIG and 1.48 t ha^−1^ in TSM in a five-year period for DK 8053 maize variety. It is, therefore, important to realise that some agroforestry technologies need a gestation period to build-up the biomass and/or nutrient before they can positively improve the soil and the resultant crop yields (Akinnifesi et al., 2006).

The *G.sepium* trimmings application in MIG might have contributed to more organic matter accumulation. Increased organic matter in soil improves water holding capacity of the soil (Brady and Weil, 1999). Good water supply and availability in soil improves crop performance. For example, Chirwa et al. (2007) found that sufficient water distribution was observed in all soil horizons in the maize-gliricidia system in December and January when maize was at vegetative stage at Makoka Research Station, southern Malawi. In the present study, the high maize yield in MIG might have also been influenced by soil moisture stability in MIG unlike in the TSM. As observed by Ong et al. (2006) in most semi-arid tropical areas, the crop stand showed water stressed crop conditions in TSM during dry spells while maize in MIG still looked unaffected by the dry spell. Dry spells were mainly experienced in the second and third seasons. *Gliricidia sepium* has a fragmentation effect in soil particles which reduces soil bulk density (Arachchi and Liyanage, 1996) and this improves and stabilises rate of water infiltration thereby contributing to good performance for the associated crop (Gunasena et al., 1991).

In a system of maize-*G. sepium*, aerial competition is reduced with consistent pruning (De Oliviera et al., 2016). Such a system allows for maximum use of resources including nutrients. *G. sepium* has less density of roots (460 cm dm^−3^) in the 0–30 cm depth while maize has 1200 cm dm^−3^ root density in the same depth (Makumba, 2003). Therefore, in a system of maize-*G. sepium* where maize produce high yields, this could mean that *G. sepium* has less competition for resources underground at least in the 0–30 cm depth where nutrients are concentrated making them accessed by the maize crop (Makumba et al., 2006).

Based on the results, it is advisable to promote agroforestry (maize-gliricidia intercropping) as a system of crop production in Salima District, central Malawi. More Gliricidia-maize intercropping fields could be set at household level. The *G.spium*-maize intercropping should be practised for a couple of years (Akinnifesi et al., 2006; Chirwa et al., 2006; Makumba et al., 2006) to realise meaningful impact of the practice since maize yield response depends on contributions of *G. sepium* to soil nutrient status. It is recommended to keep on supplementing additional N application in the system since the removal of woody materials (firewood-common under farmer conditions) of *G. sepium* in *G. sepium*-maize system, removes about 20–30 kg N ha^−1^ and an additional harvest of both, *G. sepium* and maize crop storks in *G. sepium*-maize system, removes 80–95 kg N ha^−1^ (Chirwa et al., 2006). Furthermore, easier to read/understand materials could be developed and distributed to the rural farmers for easy adaptability of the practise.

## 5 Conclusion

Soil nutrients levels in sole-crop field may be distinctly different from an agroforestry system practised for more than one year. Maize yield could better be improved if integrated with nitrogen fixing agroforestry species even at farmer management level. The maize-gliricidia intercropping agroforestry technology should further be upscaled into all areas where gliricidia or other green leaf manure producing multi-purpose trees grow favourably.

## Declarations

### Author contribution statement

H. Nyirenda: Conceived and designed the experiments; Performed the experiments; Analyzed and interpreted the data; Contributed reagents, materials, analysis tools or data; Wrote the paper.

### Funding statement

This work was supported by the Borlaug Higher Education for Agricultural Research and Development (award number BSF-G-11-00002).

### Competing interest statement

The authors declare no conflict of interest.

### Additional information

No additional information is available for this paper.
